# Multimodality imaging and 3D-printing of a thoraco-abdominal aortic aneurysm eroding into the spine

**DOI:** 10.1016/j.radcr.2022.11.020

**Published:** 2022-12-05

**Authors:** David J. Winkel, Edin Mujagic, Daniel Staub, Dorothee Harder, Jens Bremerich, Markus M. Obmann

**Affiliations:** a Clinic of Radiology and Nuclear Medicine, Basel University Hospital, University of Basel, Petersgraben 4, CH-4031, Basel, Switzerland; b Vascular Surgery, Department of Surgery, Basel University Hospital, University of Basel, Basel, Switzerland; c Department of Angiology, University Hospital Basel, University of Basel, Basel, Switzerland

**Keywords:** Computed tomography angiography, Magnetic resonance imaging, Printing, Three-dimensional, Stents, Follow-Up Studies

## Abstract

A rare case of a previously treated thoraco-abdominal aortic aneurysm eroding into the thoracic spine is described. Initially, several follow-up CT angiography scans showed an increasing aneurysm sack, but no endoleak could be depicted. Then, a new rapidly developing erosion into the thoracic spine was noted. MRI imaging excluded any other underlying infectious or malignant process. Additional contrast-enhanced ultrasound excluded an endoleak. A 3D-printed model of the aneurysm and spine and cinematic renderings were created to improve visualization. She underwent relining of the thoracic stent graft. Follow-up imaging showed a stable aneurysm size and no progression of the vertebral erosions.

## Introduction

An aneurysm of the descending thoracic aorta is commonly defined as a dilatation of the vessel diameter greater than 4 cm [1]. Especially in the aging population these aneurysms are a significant cause of morbidity and mortality [2]. In rare cases, aortic aneurysms have been reported to erode into the lumbar or thoracic spine [3]. Reported causes can be contained rupture, infection or inflammation [4], [5], [6]. Diagnosis and follow-up is usually made by CT angiography (CTA) [7]. Treatment options include - depending on the extent of the osseous erosion - conservative treatment, interventional stent graft placement, open surgery and combined approaches [8]. Recently 3D-printing has solidified its position to facilitate better understanding of pathologies and aid patient information and help guide treatment decisions [9], [10], [11].

## Case report

Written informed consent for the publication of this case report was obtained from the patient. A 71-year-old white female received regular follow-up CTA of a thoraco-abdominal aneurysm, which was previously treated with combined surgical and interventional approach. The ascending aorta and aortic arch were replaced with a frozen elephant trunk 4 years earlier and the descending aorta subsequently treated endovascularly with a thoracic stent graft (TEVAR) and a 2 times branched abdominal stent graft (BREVAR). Two months earlier, she had undergone a chest CT, which had shown a progressively enlarging thoraco-abdominal aneurysm sack.

**Fig. 1:**
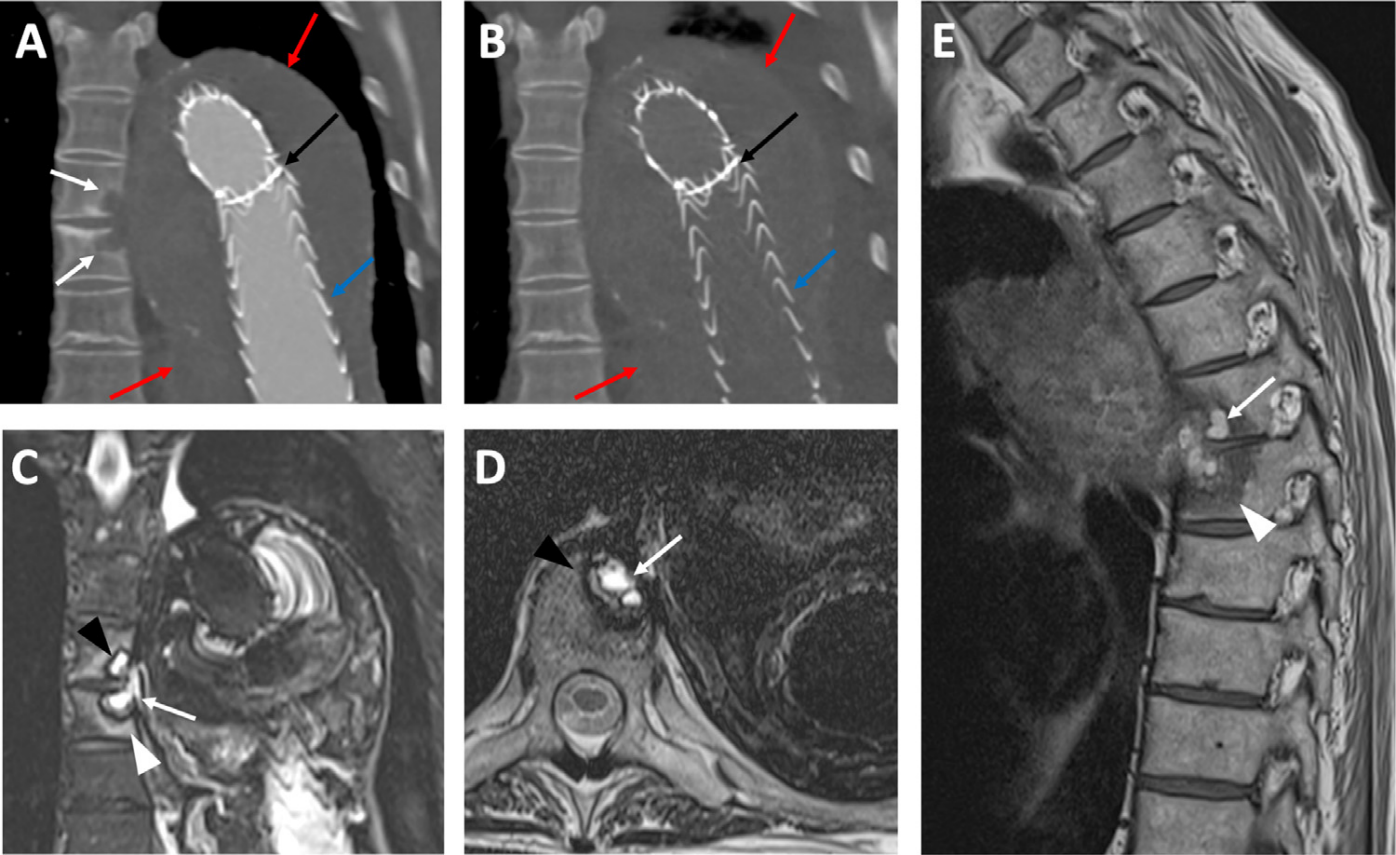
Coronal reconstruction of a regular follow-up CTA after surgical and endovascular treatment of a thoraco-abdominal aneurysm (A). Stent grafts placed into the thoraco-abdominal aneurysm (blue arrow), overlapping areas of 2 stent grafts (black arrow). New erosions of the sixth and seventh thoracic vertebra are noted (white arrows). Non-contrast CT Chest (B) 2 months prior to A shows no vertebral erosions. MRI performed 3 weeks after CT of image B (C-E): T2 TIRM-weighted coronal image (C) shows the known vertebral erosion (white arrow), with a T2- and T1-weighted hyperintense Signal (D, E), corresponding to internal hemorrhage. The erosion shows a T2-/T1-weighted hypointense rim (black arrowhead), corresponding to hemosiderin deposits (D). Surrounding T2-weighted hyperintense and T1w-hypointense edema in both vertebrae (white arrowhead) (C-E).

**Fig. 2:**
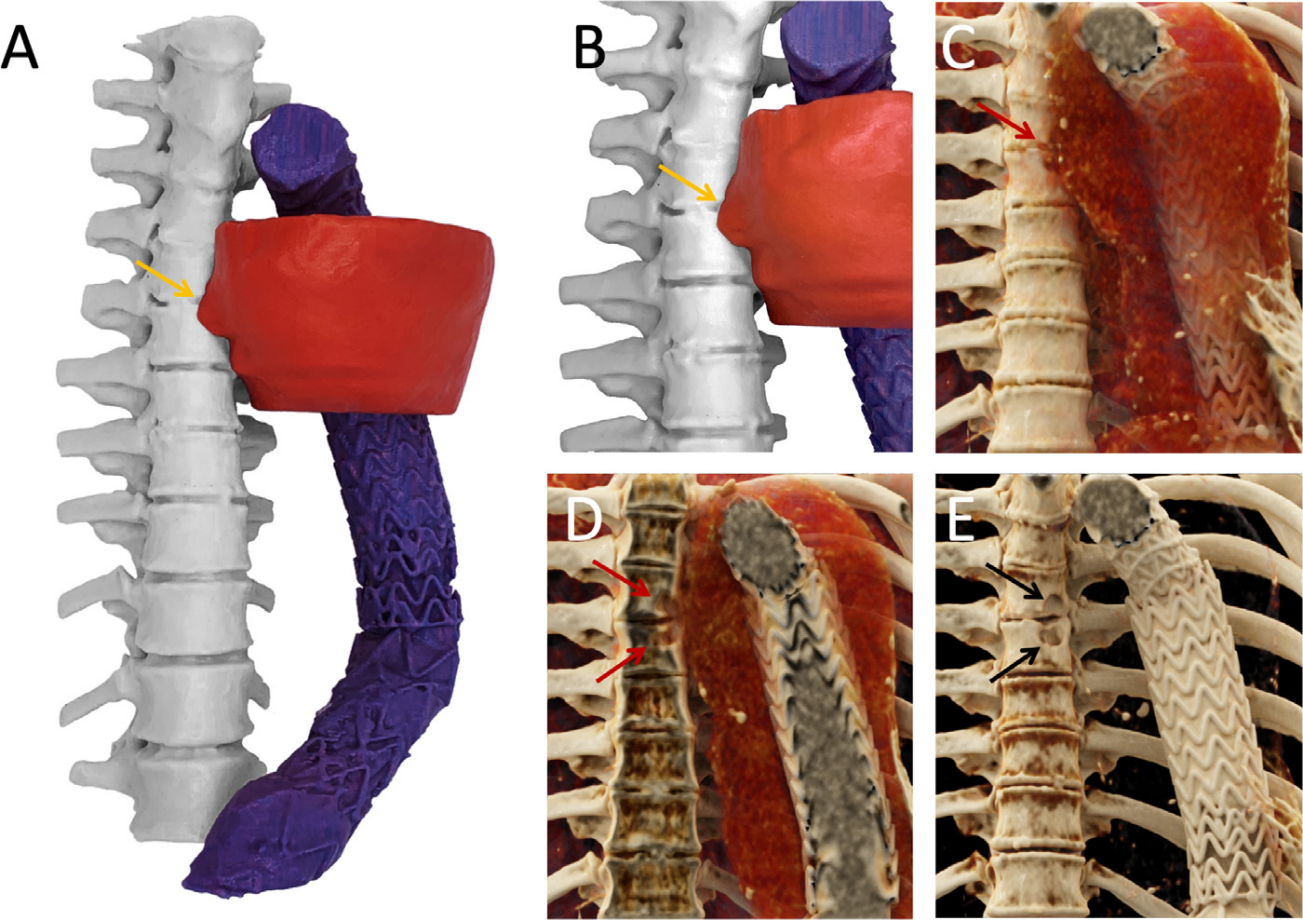
Multicolor 3-dimensional printed model (A and B) visualizing the thoracic spine (white), part of the excluded aneurysm sack (red) and stent graft (blue). The focal protrusion and erosion into the spine are clearly visible (yellow arrow). Cinematic rendering (C-E) of the aneurysm. In C the aneurysm sack (red) is shown as translucent. The focal protrusion and erosion into the spine is shown similarly to the 3D-printed model (A and B). In D the cinematic rendered volume of C is cut in a coronal plane revealing more detailed how the aneurysm sack erodes into the vertebral bodies (red arrows). Cinematic rendering of the high-density structures in E further points out the osseous defects (black arrows).

This follow-up exam revealed a new erosion of the sixth and seventh thoracic vertebra adjacent to the further enlarging thoraco-abdominal aneurysm, not present 2 months earlier (Figs. 1A and B). The erosion was suspected to be pressure-based due to the enlarging aneurysm sack. A model of the thoracic spine, excluded aneurysm sack and stent graft was 3D-printed based on the CT dataset (Fig. 2). Binder jet technology (ProJet 660 Pro, 3D Systems, Rock Hill, USA) and white composite as the primary material (PXL VisiJet, 3D Systems, Rock Hill, USA) was used. After semi-automated segmentation, a model was created using dedicated software (Mimics Medical 24.0, Materialise, Leuven, Belgium and 3-matic Medical 16.0 (Materialise, Leuven, Belgium). The printed model was post-processed using a synthetic resin (ColorBond Instant Infiltrant, 3D Systems, Rock Hill, USA), increasing hardness and strengthening color.

Operative, interventional and conservative treatment options were discussed with the patient. As concomitant disease, the patient suffered from achalasia and was cachectic due to impaired food intake. This was recently treated with botulinum injections and the patient's overall condition was just beginning to improve. Therefore, a final treatment decision was deferred for another 6 weeks to wait for an improvement of the overall condition of the patient. In the meantime, an MRI was planned and obtained 3 weeks later to rule out any infection or malignant origin. The MRI showed further enlarging erosions of the vertebrae, with central hemorrhage and hemosiderin depositions on the edge, but no other reason of the erosion (Figs. 1C-E).

**Fig. 3:**
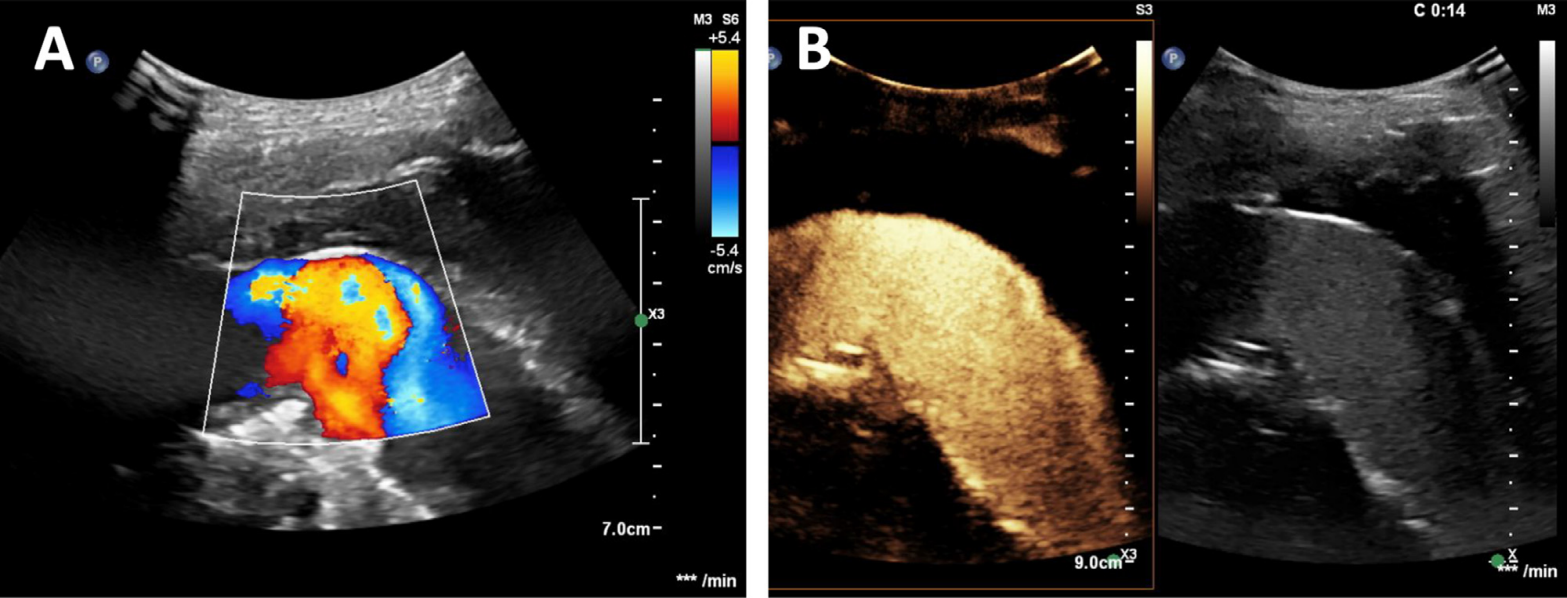
Color-coded Doppler ultrasound (A) shows flow in the stented aneurysm but no flow in the excluded aneurysm sac. Contrast-enhanced ultrasound (B) also shows no perfusion of the excluded aneurysm sack.

As the aneurysm sack was growing further, a contrast-enhanced ultrasound (CEUS) and color Doppler ultrasound were also performed using a transcostal view based on an ideal anatomy to detect an endoleak responsible for the aneurysm growth. No endoleak could be visualized (Figs. 3A and B). The overlapping ends of the stent grafts showed pulsation synchronous fabric movements as a theoretically increased risk for an endoleak. Two weeks after the initial presentation the patient was shortly hospitalized for 2 days because of severe abdominal pain due to obstipation and stercoral colitis which was treated successfully with laxatives.

As MRI had ruled out any other reason of the erosions, ultrasound and CTAs did not demonstrate any direct endoleak (type I-III) [12], but the aneurysm sack had been growing, clinically endotension was suspected. Factoring in the risk for perforation and the progressive spinal erosion, as well as the multiple comorbidities of the patient, a decision was reached to reline the thoracic stent grafts. Starting at the elephant trunk a Zenith TX 2 graft (ZDEG-PT-32-202-PF, Cook Medical, Bloomington, IN) was placed into the previous stent graft and adjusted with a balloon, followed distally by another graft of the same sizing, ending just proximally to the abdominal EVAR branches. Postoperative follow-up CTA the next day showed no endoleak and a normal perfusion of the stent grafts. Two-month follow up also showed no endoleak, aneurysm growth or progressive erosions.

## Discussion

Descending thoracic aortic aneurysms are a common cause of morbidity and mortality in the aging population. An endovascular approach is now the preferred way of treatment for most cases [13]. Follow-up exams are usually performed with CTA [7]. Erosion of the aneurysm into the thoracic spine is a rare complication, even more so when the aneurysm has already been treated [8]. Persistent aneurysm growth and vertebral erosions can often be asymptomatic and are therefore only noticed on regular follow-up exams.

As shown in the case presented here, besides CTA for the initial diagnosis, other imaging modalities also play a crucial role to assess complications and causes. MRI imaging is helpful to exclude another underlying malignant or infectious process of the spinal erosion. CEUS is highly sensitive for the detection of endoleaks [14].

The choice of treatment depends on the extent of the spinal erosion, aneurysm growth, as well as patient comorbidities. Here advanced visualization techniques can be employed. 3D printing and photo-realistic cinematic rendering are excellent tools to demonstrate such complex pathologies to the patients affected, who are not familiar with advanced vascular imaging. As such, true patient informed consent and decision making can be achieved.

## Conclusion

In this case, we used the state-of-the art imaging methods of CT, Ultrasound and MRI in the precise detection and differential diagnosis of a bony erosion due to a thoraco-abdominal aneurysm. Novel methods for visualization including a multi-colored 3D-printed model and cinematic rendering further enhance visuospatial appreciation of this pathology and patient informed decision-making.

## Patient consent

Informed consent for publication was obtained.
